# Initial validation of the Hungarian Eating Disorder Examination Questionnaire Short (EDE-QS-H) in a sample of recreational athletes

**DOI:** 10.3389/fnut.2026.1943893

**Published:** 2026-10-07

**Authors:** Réka Erika Kovács, Katarina Prnjak, Sohom Saha, İsmail İlbak, Szilvia Boros, Gusztáv József Tornóczky

**Affiliations:** 1 Department of Psychology and Health Management, Faculty of Health and Sport Sciences, Széchenyi István University, Gyor, Hungary; 2 Graduate School of Health, University of Technology Sydney, Sydney, NSW, Australia; 3 Department of Sport Psychology, Lakshmibai National Institute of Physical Education, Gwalior, India; 4 Faculty of Sports Sciences, İnönü University, Malatya, Türkiye; 5 Institute of Health Promotion and Sport Sciences, Faculty of Education and Psychology, ELTE Eötvös Loránd University, Budapest, Hungary; 6 Physical Education and Exercise Centre, Medical School, University of Pécs, Pécs, Hungary

**Keywords:** athletes, confirmatory factor analysis, eating disorder, EDE-QS-H, psychometrics, questionnaire validation

## Abstract

**Introduction:**

Eating disorders represent an important health concern among athletes, yet validated Hungarian screening tools remain limited. The present study aimed to translate, culturally adapt, and psychometrically validate the Hungarian version of the Eating Disorder Examination Questionnaire–Short (EDE-QS-H) in a sample of Hungarian athletes. A secondary aim was to examine the relationship between eating disorder psychopathology and multidimensional self-reported interoceptive awareness.

**Methods:**

A total of 205 recreational athletes recruited from two university settings (51.7% women; mean age = 21.8 ± 5.6 years) completed the Hungarian EDE-QS, the Disordered Eating Screen for Athletes–Hungarian version (DESA-6H), and the Hungarian Multidimensional Assessment of Interoceptive Awareness (MAIA). Confirmatory factor analysis (CFA) was performed using the robust weighted least squares estimator with mean and variance adjustment (WLSMV). Internal consistency was evaluated using Cronbach’s *α* and McDonald’s *ω*, whereas construct validity was assessed using Pearson correlation coefficients.

**Results:**

Sequential psychometric refinement resulted in a 10-item unidimensional model with improved model fit (*χ*^2^ = 82.77, df = 35, *χ*^2^/df = 2.37, CFI = 0.987, TLI = 0.984, RMSEA = 0.082, SRMR = 0.066). The RMSEA was marginally above the conventional 0.08 criterion, whereas the remaining indices supported an acceptable measurement model. Standardized factor loadings ranged from 0.660 to 0.925, and the Average Variance Extracted was 0.671. The final version demonstrated excellent internal consistency (Cronbach’s *α* = 0.902; McDonald’s *ω* = 0.942). Construct validity was supported by a strong positive correlation with the DESA-6H (*r* = 0.643, *p* < 0.001), while associations with MAIA subscales were heterogeneous and generally small, indicating dimension-specific rather than broad relationships with self-reported interoceptive awareness.

**Conclusion:**

This initial validation provides evidence that the refined 10-item EDE-QS-H has promising factorial validity, excellent internal consistency, and construct-validity support in this university-based sample of Hungarian recreational athletes. The instrument may be useful as a brief research and preliminary screening measure of eating disorder psychopathology; however, diagnostic validity and broader clinical utility require evaluation in independent and clinical samples.

## 1 Introduction

Eating disorders (EDs) pose a significant public health challenge, with their burden well established in the literature (1, 2). Clinical EDs, including anorexia nervosa (AN), bulimia nervosa (BN), and binge eating disorder (BED), are diagnostically defined conditions, whereas disordered eating (DE) refers more broadly to maladaptive eating and weight-control behaviors that may occur below diagnostic thresholds. Eating disorder psychopathology encompasses the cognitive and behavioral features underlying these conditions, including excessive concerns about body weight and shape, restrictive eating, and overvaluation of shape or weight (3, 4). Because ED symptoms occur along a continuum and subclinical manifestations are common in the general population (5, 6), valid and practical screening instruments are important for identifying elevated symptom burden or risk warranting further assessment.

One of the most widely used instruments for assessing ED psychopathology is the Eating Disorder Examination Questionnaire (EDE-Q), a self-report measure derived from the Eating Disorder Examination interview. Although the EDE-Q provides a comprehensive assessment of ED-related attitudes and behaviors, its length may limit its feasibility in research settings where multiple constructs are assessed or participant burden is a concern. To address this limitation, Gideon et al. (7) developed the 12-item Eating Disorder Examination Questionnaire Short Form (EDE-QS), a brief measure designed to assess overall ED psychopathology over the preceding 7 days. Subsequent research has supported its unidimensional structure and utility as a brief measure of ED symptom severity (8). However, prior to the present study, no validated Hungarian adaptation of the EDE-QS was available.

The availability of a brief Hungarian measure may be particularly relevant for physically active populations. Although EDs have traditionally been investigated primarily in clinical populations, increasing evidence indicates that DE and elevated ED psychopathology also occur among athletes and physically active individuals (9, 10). Sport participation may involve pressures related to body composition, weight management, appearance, and performance, which may increase vulnerability to maladaptive eating behaviors (11, 12). While these risks are well documented among elite athletes, recreational athletes may also represent a vulnerable population, particularly in aesthetic, endurance, and weight-sensitive sports (11). Unlike elite athletes, recreational athletes may have limited access to professional nutritional or psychological support despite exposure to similar body- and performance-related pressures (13, 14). The importance of identifying DE and EDs in physically active populations is also reflected in the relative energy deficiency in sport (REDs) framework, which recognizes that problematic eating behaviors may contribute to inadequate energy availability and associated adverse health and performance outcomes (15).

In Hungary, athlete-specific screening for DE risk is available through the DESA-6H (16). However, athlete-specific risk screening and the assessment of global ED psychopathology serve related but distinct purposes. The EDE-QS provides a concise measure of overall ED symptom severity rather than a sport-specific risk classification and may therefore complement existing athlete-specific screening instruments. Accordingly, validation of a Hungarian version of the EDE-QS addresses an important measurement gap and may facilitate its use in both research and screening contexts.

Interoceptive awareness may represent an additional construct relevant to ED psychopathology. Interoception refers broadly to the perception and interpretation of internal bodily signals, including hunger and satiety, and has been implicated in contemporary models of EDs (17–19). Individuals with ED symptoms may experience difficulties recognizing or trusting bodily sensations, which may interfere with adaptive eating regulation (20–24). In the present study, interoceptive awareness was assessed using the Hungarian version of the Multidimensional Assessment of Interoceptive Awareness (MAIA) (25, 26), which measures self-reported dimensions of bodily awareness rather than objective interoceptive accuracy. Examining its relationship with EDE-QS scores provides an opportunity to explore the association between ED psychopathology and multidimensional self-reported interoceptive awareness in recreational athletes.

Therefore, the primary aim of the present study was to adapt the EDE-QS into Hungarian and evaluate its psychometric properties in a sample of recreational athletes recruited from university settings. A secondary aim was to examine the relationship between ED psychopathology and multidimensional self-reported interoceptive awareness. We expected the Hungarian EDE-QS to demonstrate satisfactory psychometric properties and hypothesized that greater ED symptom severity would be associated with lower levels of adaptive interoceptive awareness. Given the multidimensional structure of the MAIA, these associations were examined at the subscale level.

## 2 Methods

### 2.1 Study design and ethical considerations

Ethical approval for this study was granted by the Research Ethics Committee of Széchenyi István University on August 28, 2025 (Reference No. SZE/ETT-52/2025). Following translation of the EDE-QS, the Hungarian validation and assessment of ED risk were conducted using convenience sampling. Participants were recruited from the Faculty of Health and Sports Sciences at Széchenyi István University and the Faculty of Education and Psychology at Eötvös Loránd University; accordingly, the sample represents recreational athletes from university settings rather than the broader Hungarian athlete population. Participation was voluntary, and no financial or other compensation was provided.

For the purposes of the present study, the inclusion criteria were: (1) age ≥18 years; (2) current, regular participation in leisure-time physical activity for at least 150 min per week; and (3) regular participation in and specification of a sport. Recreational athletes were operationally defined as adults who regularly participated in a self-reported sport for recreational rather than professional or elite competitive purposes. The 150-min-per-week criterion was used as an eligibility threshold to ensure regular engagement in physical activity (27). Accordingly, the study specifically focused on sport-engaged recreational athletes; individuals whose physical activity consisted exclusively of non-sport activities, such as walking or group exercise classes without participation in a specific sport, were outside the target population.

Of the 303 responses received, individuals were excluded if they provided incomplete responses (*n* = 51), did not meet the eligibility criteria for regular physical activity (i.e., <150 min per week; *n* = 34), or did not specify the sport in which they participated (*n* = 13), resulting in a final sample of 205 participants. Electronic informed consent was obtained before questionnaire access by requiring respondents to confirm consent via a checkbox after scanning a QR code. Data collection was conducted entirely online using a self-administered format. The same sample was used for the validation of the Hungarian Eating Disorder Examination Questionnaire–Short and for examining associations between ED psychopathology and self-reported interoceptive awareness.

### 2.2 Participant characteristics

A total of 205 athletes participated in the study. The mean age of the participants was 21.8 years (SD = 5.6). The sample comprised 99 men (48.3%) and 106 women (51.7%). On average, participants had been practicing their primary sport for 10.4 years (SD = 5.7). Participants represented a broad range of sports. The largest proportion competed in team sports (*n* = 95, 46.3%), followed by aesthetic sports (*n* = 37, 18%), strength-endurance sports (*n* = 25, 12.1%), strength sports (*n* = 19, 9.3%), weight-dependent sports (*n* = 15, 7.3%), and endurance sports (*n* = 13, 6.3%). Finally, only one participant represented a technical sport (0.5%).

Regarding weekly training volume, 90 participants (43.9%) trained 3–6 h/week, 63 (30.7%) trained 7–10 h/week, 28 (13.7%) trained 11–14 h/week, and 24 (11.7%) reported training more than 14 h/week.

Given that the EDE-QS comprises 12 items, the present sample yielded a participant-to-item ratio of approximately 17:1. This exceeds commonly recommended sample size guidelines for psychometric validation and factor-analytic studies, which generally suggest a minimum of 5–10 participants per item, with at least 200 participants considered adequate for stable factor solutions and questionnaire validation (28, 29).

### 2.3 Eating disorder examination (EDE)

The Eating Disorder Examination (EDE), developed by Cooper and Fairburn (30), was originally introduced as a semi-structured clinical interview for the comprehensive assessment of eating disorder psychopathology and remains the gold standard for both clinical diagnosis and research. To facilitate large-scale epidemiological and clinical studies, Fairburn and Beglin (31) developed the Eating Disorder Examination Questionnaire (EDE-Q), a self-report version that retained the four original subscales (Restraint, Eating Concern, Shape Concern, and Weight Concern). The most widely used version, the EDE-Q 6.0, comprises 28 items assessing eating disorder symptoms over the preceding 28 days (32). Evidence supports the validity of the EDE-Q as a screening instrument in community samples (33), and normative data have also been established for specific populations, including clinical samples of males (34).

In order to reduce respondent burden while maintaining satisfactory psychometric performance, several abbreviated versions of the EDE-Q have been developed. Among these, the Eating Disorder Examination-Questionnaire 8 (EDE-Q8) is an 8-item short form that provides a brief assessment of global eating disorder psychopathology while retaining good psychometric properties, making it particularly suitable for screening and large-scale epidemiological research (35). Another widely used abbreviated version is the Eating Disorder Examination Questionnaire Short (EDE-QS), developed by Gideon et al. (7). The EDE-QS is a 12-item unidimensional measure designed to assess the overall severity of eating disorder psychopathology rather than distinct symptom domains. Unlike the original EDE-Q, it does not generate subscale scores; instead, responses are summed to produce a single total score ranging from 0 to 36, with higher scores indicating greater symptom severity. Furthermore, the EDE-QS assesses symptoms experienced during the previous 7 days, making it particularly suitable for repeated assessments and monitoring symptom change over time. In the initial validation study, the EDE-QS demonstrated excellent internal consistency (Cronbach’s *α* = 0.91), high test–retest reliability, and strong convergent validity with the original EDE-Q and other established measures of eating disorder psychopathology (7). Subsequently, Prnjak et al. (8) confirmed its unidimensional structure and screening utility and recommended a cut-off score of 15, which provided the optimal balance between sensitivity (0.83) and specificity (0.85) for identifying probable eating disorder cases in community samples. Accordingly, the present study employed the Prnjak et al. (8) version of the EDE-QS because of its robust psychometric support, established screening performance, and suitability for assessing overall eating disorder psychopathology in repeated measurements.

### 2.4 Development of the Eating Disorder Examination Questionnaire—Short Hungarian version

Permission to adapt the original questionnaire was acquired on June 12, 2025. In accordance with the questionnaire adaptation methodology outlined by Beaton et al. (36), two experts independently translated the questionnaire into Hungarian, and discrepancies were resolved through consensus. Following this, an independent expert, who had not seen the original version, conducted a back-translation into English. The resulting English version was then sent to the original author for review, and the Hungarian version was refined based on the feedback received.

### 2.5 Multidimensional Assessment of Interoceptive Awareness

The Multidimensional Assessment of Interoceptive Awareness (MAIA) was developed by Mehling et al. (37) as a self-report instrument designed to assess multiple dimensions of interoceptive body awareness. Unlike earlier measures that conceptualized interoception as a unitary construct, the MAIA evaluates eight theoretically derived domains: Noticing, Not-Distracting, Not-Worrying, Attention Regulation, Emotional Awareness, Self-Regulation, Body Listening, and Trusting. The original instrument consists of 32 items rated on a 6-point Likert scale ranging from 0 (“never”) to 5 (“always”), with higher scores indicating greater interoceptive awareness in the respective domain. In the original validation study, the subscales demonstrated acceptable to good internal consistency, with Cronbach’s *α* coefficients ranging from 0.66 to 0.87, and the proposed eight-factor structure was supported (37). Two Hungarian adaptations of the MAIA have been published (25, 26). Járai et al. (25) translated and validated the original 32-item MAIA in a Hungarian adult sample, confirming the multidimensional factor structure and reporting satisfactory internal consistency for the Hungarian subscales (Cronbach’s *α* ranging from 0.72 to 0.90). Subsequently, Ferentzi et al. (26) adapted the revised MAIA-2, which includes additional items to improve the reliability of the Not-Distracting and Not-Worrying subscales. In the present study, the Hungarian version developed by Járai et al. (25) was used.

### 2.6 Disordered Eating Screen for Athletes (DESA-6)

The Disordered Eating Screen for Athletes (DESA-6) was developed by Kennedy et al. (38) as a brief screening instrument specifically designed to identify athletes at risk for disordered eating. Unlike generic eating disorder questionnaires, the DESA-6 incorporates sport-specific risk factors and is applicable to both male and female athletes across a wide range of sports. The instrument consists of six items assessing weight-related concerns, dieting behaviors, injury history, perceived pressure to lose weight, and other sport-specific risk factors. Responses are summed to yield a total score ranging from 0 to 9, with higher scores indicating a greater risk of disordered eating. A score of ≥3 has been recommended as the screening cut-off. In the original validation study, the DESA-6 demonstrated excellent screening performance against the Eating Disorder Examination (EDE 17.0D), with 92.0% sensitivity and 86.0% specificity, supporting its use as a rapid screening instrument for athletes (38). The Hungarian version (DESA-6H) was translated and culturally adapted by Kovács et al. (16). Its convergent validity was supported by a moderate positive correlation with the EAT-26 total score (Kendall’s *τ*_*b* = 0.49, *p* < 0.001), as well as with the Dieting (*τ*_*b* = 0.53, *p* < 0.001) and Bulimia (*τ*_*b* = 0.39, *p* < 0.001) subscales. Furthermore, the Hungarian version demonstrated good diagnostic accuracy for identifying athletes at risk of disordered eating (AUC = 0.82, 95% CI = 0.71–0.93, *p* < 0.001), supporting its applicability as a screening tool in Hungarian athletic populations (16). Accordingly, the Hungarian DESA-6H was used in the present study.

### 2.7 Statistical analysis

Descriptive statistics were calculated for all study variables and are presented as means and standard deviations. The distributions of the study variables were evaluated using skewness and kurtosis to assess univariate normality and to inform the choice of correlation coefficients. Across all measures, skewness ranged from −0.736 to 1.249 and kurtosis ranged from −0.079 to 0.783, indicating that the distributions were within the recommended limits for univariate normality (39).

The factorial validity of the Hungarian version of the Eating Disorder Examination Questionnaire–Short (EDE-QS-H) was evaluated using confirmatory factor analysis (CFA). Because the EDE-QS consists of ordinal Likert-type items, CFA models were estimated using the robust weighted least squares estimator with mean and variance adjustment (WLSMV), which is recommended for ordinal indicators because it is based on polychoric correlations and does not assume multivariate normality (40, 41). A sequential model-refinement strategy was applied. Item retention was evaluated using standardized factor loadings, overall model fit, modification indices, and the conceptual content represented by each item. Items were considered for removal only when statistical evidence indicated inadequate measurement performance, such as a low standardized factor loading or substantial local dependency, and when the remaining items continued to represent the core domains of global eating disorder psychopathology assessed by the EDE-QS. Thus, item removal was not based solely on improvements in statistical model fit but also considered the potential impact on the content coverage of the construct. Because these modifications were derived in the same sample in which model fit was evaluated, the refined solution was treated as provisional and requires replication in an independent sample to evaluate its stability and the potential for sample-specific overfitting.

Model fit was evaluated using the chi-square statistic (*χ*^2^), the chi-square to degrees of freedom ratio (*χ*^2^/df), the Comparative Fit Index (CFI), the Tucker–Lewis Index (TLI), the Root Mean Square Error of Approximation (RMSEA) with its 90% confidence interval, and the Standardized Root Mean Square Residual (SRMR). Consistent with widely accepted recommendations, CFI and TLI values ≥0.95 were considered indicative of excellent model fit, whereas RMSEA and SRMR values ≤0.08 were considered indicative of acceptable fit (40–42).

Internal consistency was evaluated using both Cronbach’s *α* and McDonald’s *ω* coefficients, with values ≥0.70 considered acceptable (43–45). Internal convergent validity was additionally assessed using the Average Variance Extracted (AVE), for which values ≥0.50 were considered indicative of adequate convergent validity (46).

Construct validity was examined by calculating Pearson product–moment correlation coefficients between the EDE-QS-H total score, the Disordered Eating Screen for Athletes (DESA-6H), and the subscales of the Multidimensional Assessment of Interoceptive Awareness (MAIA). Pearson correlations were selected because the distributions of the study variables met the recommended criteria for univariate normality (39). Correlation coefficients of approximately 0.10, 0.30, and 0.50 were interpreted as small, moderate, and large effect sizes, respectively (47). For key correlations, 95% confidence intervals were reported to provide information on the precision of the estimated associations and to support interpretation of their practical magnitude.

All descriptive statistics and correlation analyses were conducted using IBM SPSS Statistics (Version 31; IBM Corp., Armonk, NY, USA), whereas confirmatory factor analyses were performed using JASP (Version 0.95.4; JASP Team).

## 3 Results

### 3.1 Descriptive statistics

Descriptive statistics for the Hungarian EDE-QS indicated a mean total score of 5.35 (SD = 6.00), with scores ranging from 0 to 25 (maximum possible score = 30; median = 3.00). In the sex-based comparison, female participants had higher mean scores (7.35 SD = 6.85) than male participants (3.19 SD = 3.94). The distribution of the total score showed moderate positive skewness (skewness = 1.24) and acceptable kurtosis (0.77), reflecting the predominance of lower symptom levels in this non-clinical sample. No missing data were observed for the EDE-QS-H total score (*N* = 205).

### 3.2 Confirmatory factor analysis (CFA) and psychometric refinement

To evaluate the factorial validity of the Hungarian version of the Eating Disorder Examination Questionnaire–Short (EDE-QS), a series of one-factor confirmatory factor analyses (CFA) was conducted using the WLSMV estimator, which is appropriate for ordinal Likert-type indicators. Model refinement was performed sequentially based on standardized factor loadings, overall model fit, and inspection of modification indices while preserving the conceptual integrity of the original instrument. The fit indices for all three sequential versions of the scale are presented in Table 1.

**Table 1:** Confirmatory factor analysis (CFA) fit indices for the sequential refinement of the Hungarian EDE-QS.

Model	Items	*χ* ^2^	df	*χ*^2^/df	CFI	TLI	RMSEA (90% CI)	SRMR	Decision
Model 1	12	165.30	54	3.06	0.971	0.965	0.100 (0.083–0.118)	0.088	EDE12 removed (*λ* = 0.251)
Model 2	11	154.10	44	3.50	0.972	0.965	0.111 (0.092–0.130)	0.093	EDE10 removed (local dependency)
Model 3	10	82.77	35	2.37	0.987	0.984	0.082 (0.059–0.105)	0.066	Final model

CFA, confirmatory factor analysis; CFI, Comparative Fit Index; TLI, Tucker–Lewis Index; RMSEA, Root Mean Square Error of Approximation; SRMR, Standardized Root Mean Square Residual. Model refinement was based on standardized factor loadings and inspection of modification indices while preserving the conceptual integrity of the original scale.

The initial 12-item version demonstrated acceptable incremental fit indices (CFI = 0.971, TLI = 0.965); however, the RMSEA (0.100) and SRMR (0.088) exceeded commonly recommended thresholds, indicating room for model improvement. Examination of standardized factor loadings revealed that Item EDE12, assessing dissatisfaction with body weight or shape, exhibited a substantially weaker association with the latent construct (*λ* = 0.251) than the remaining indicators. Given its weak factor loading, EDE12 was considered for removal. From a content perspective, its removal reduces the explicit coverage of body- and weight-related dissatisfaction; however, related weight- and shape-focused psychopathology remained represented by several retained items addressing weight/shape preoccupation, fear of weight gain, desire for weight loss, and the influence of appearance or body weight on self-image. Thus, the removal of EDE12 was considered consistent with both its psychometric performance and the content coverage of the retained item set.

The revised 11-item version retained satisfactory CFI and TLI values but continued to demonstrate suboptimal absolute fit, particularly for RMSEA (0.111) and SRMR (0.093). Modification indices indicated substantial localized strain involving Item EDE10, which asks about episodes involving an unusually large amount of food on occasions when loss of control was experienced. Although EDE10 captures the objective quantity aspect of binge eating, the retained Item EDE9 directly assesses loss of control over eating, representing the central feature of this domain. Removal of EDE10 therefore reduces the specific coverage of objective binge-eating quantity but preserves the core loss-of-control component within the global eating disorder psychopathology construct. EDE10 was consequently removed based on the combination of local-dependency evidence, its overlap with the retained loss-of-control item, and consideration of the resulting content coverage, rather than statistical model improvement alone.

The final 10-item version (Supplementary Table S1) demonstrated a mixed but substantially improved fit profile (Table 1): CFI (0.987) and TLI (0.984) indicated excellent incremental fit, and SRMR (0.066) was acceptable, whereas RMSEA [0.082; 90% CI (0.059, 0.105)] was marginally above the conventional 0.08 criterion. Accordingly, the final model was interpreted as providing acceptable-to-good overall support rather than uniformly excellent fit. The sequential modifications were data-informed and should be cross-validated in an independent sample before the 10-item structure is considered stable across populations.

Standardized factor loadings for the final 10-item model are presented in Table 2 and illustrated in Figure 1. All items loaded significantly on the latent factor (*p* < 0.001), with standardized loadings ranging from 0.660 to 0.925. The strongest loadings were observed for Items EDE3, EDE4, EDE5, and EDE6 (*λ* = 0.904–0.925), whereas Item EDE7 demonstrated the lowest, yet still acceptable, loading (*λ* = 0.660). Explained variance (*R*^2^) ranged from 0.435 to 0.856, indicating that all retained items contributed meaningfully to the measurement of the underlying construct.

**Table 2:** Standardized factor loadings, explained variance, reliability, and convergent validity indices for the final 10-item Hungarian version of the Eating Disorder Examination Questionnaire–Short (EDE-QS-H).

Item	Standardized factor loading (*λ*)	Explained variance (*R*^2^)
EDE1	0.804	0.646
EDE2	0.718	0.515
EDE3	0.925	0.856
EDE4	0.924	0.854
EDE5	0.904	0.818
EDE6	0.914	0.835
EDE7	0.660	0.435
EDE8	0.785	0.616
EDE9	0.801	0.642
EDE11	0.704	0.496
Scale-level psychometric indices		
Cronbach’s *α*	0.902	
McDonald’s *ω*	0.942	
Average Variance Extracted (AVE)	0.671	

*λ*, standardized factor loading; *R*^2^, explained variance of each observed indicator; AVE, Average Variance Extracted. All standardized factor loadings were statistically significant (*p* < 0.001). Cronbach’s *α* and McDonald’s *ω* indicate excellent internal consistency, whereas the AVE exceeded the recommended threshold of 0.50, supporting the convergent validity of the final 10-item Hungarian EDE-QS.

**Figure 1:**
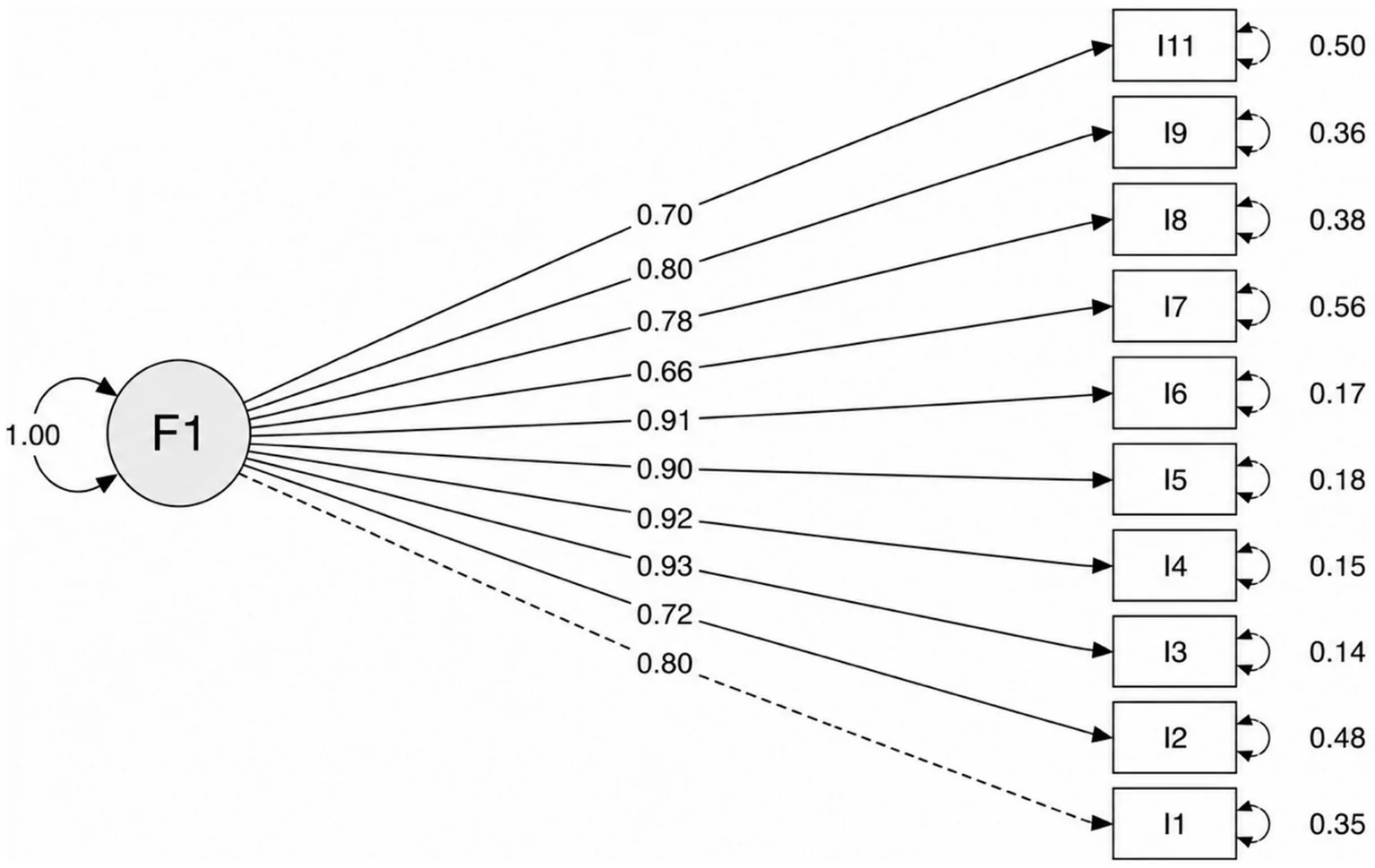
Final one-factor confirmatory factor analysis (CFA) model of the Hungarian EDE-QS.

The final Hungarian EDE-QS also demonstrated excellent internal consistency, with a Cronbach’s *α* of 0.902 and a McDonald’s *ω* of 0.942. Furthermore, the Average Variance Extracted (AVE = 0.671) exceeded the recommended threshold of 0.50, providing additional evidence for the convergent validity of the final one-factor solution (Table 2).

### 3.3 Construct validity

Construct validity of the Hungarian EDE-QS was evaluated by examining its associations with the Disordered Eating Screen for Athletes (DESA-6H) and the subscales of the Multidimensional Assessment of Interoceptive Awareness (MAIA) using Pearson product–moment correlation coefficients (Table 3).

**Table 3:** Construct validity of the Hungarian EDE-QS: Pearson correlations with the DESA-6H and MAIA subscales (*N* = 205).

Measure	M	SD	*r* with EDE-QS-H	95% CI	*p*
DESA-6H	1.39	1.84	0.643	[0.555, 0.717]	<0.001
MAIA					
Noticing	2.78	1.24	0.151	[0.014, 0.282]	0.031
Not-Distracting	2.80	1.09	−0.229	[−0.355, −0.095]	<0.001
Not-Worrying	2.57	0.98	−0.290	[−0.411, −0.159]	<0.001
Attention Regulation	2.82	1.00	−0.021	[−0.158, 0.116]	0.767
Emotional Awareness	3.03	1.13	0.104	[−0.034, 0.238]	0.139
Self-Regulation	2.59	1.13	−0.109	[−0.242, 0.029]	0.120
Body Listening	2.60	1.12	−0.019	[−0.156, 0.119]	0.790
Trusting	3.50	1.19	−0.233	[−0.359, −0.099]	<0.001

M, mean; SD, standard deviation; *r*, Pearson product–moment correlation coefficient; CI, confidence interval. Positive coefficients indicate that higher EDE-QS-H scores were associated with higher scores on the corresponding construct, whereas negative coefficients indicate inverse associations. DESA-6H, Disordered Eating Screen for Athletes—Hungarian version; MAIA, Multidimensional Assessment of Interoceptive Awareness.

The Hungarian EDE-QS demonstrated a large positive correlation with the DESA-6H (*r* = 0.643, *p* < 0.001), providing strong convergent-validity evidence between two conceptually related measures of disordered eating. Associations with the MAIA subscales were substantially smaller. Not-Worrying showed a small-to-moderate inverse association (*r* = −0.290, *p* < 0.001), whereas Not-Distracting (*r* = −0.229, *p* < 0.001) and Trusting (*r* = −0.233, *p* < 0.001) showed small inverse associations. Noticing showed a small positive correlation (*r* = 0.151, *p* = 0.031). Although several MAIA associations reached statistical significance, their small effect sizes indicate that these relationships should not be interpreted as broadly or practically substantial.

In contrast, correlations with Attention Regulation (*r* = −0.021, *p* = 0.767), Emotional Awareness (*r* = 0.104, *p* = 0.139), Self-Regulation (*r* = −0.109, *p* = 0.120), and Body Listening (*r* = −0.019, *p* = 0.790) were negligible and non-significant. Overall, the MAIA findings provide only partial support for the hypothesized associations and suggest that the relationship between eating disorder psychopathology and self-reported interoceptive awareness is dimension-specific rather than generalized across interoceptive dimensions.

## 4 Discussion

The present study translated, culturally adapted, and conducted an initial psychometric validation of the Hungarian version of the Eating Disorder Examination Questionnaire–Short (EDE-QS-H) in a university-based sample of Hungarian recreational athletes. The findings provide promising, but not definitive, support for the adapted instrument. Specifically, the analyses supported a refined 10-item (Supplementary Table S1) unidimensional structure with excellent internal consistency, strong convergence with the DESA-6H, and smaller, heterogeneous associations with selected dimensions of self-reported interoceptive awareness. These findings extend previous validation work by providing initial evidence for use of the EDE-QS-H in Hungarian recreational athletes, while independent replication remains necessary.

The psychometric characteristics of the Hungarian version were largely consistent with previous validation studies of the original EDE-QS. The excellent internal consistency observed in the present study (Cronbach’s *α* = 0.902; McDonald’s *ω* = 0.942) closely resembles the reliability reported by Gideon et al. (7), while the unidimensional structure agrees with the recommendations of Prnjak et al. (8), who demonstrated that the EDE-QS provides a brief yet comprehensive measure of global eating disorder psychopathology. Together, these findings suggest that the questionnaire retains its measurement properties despite linguistic and cultural adaptation, supporting its applicability beyond the population in which it was originally developed (7, 8).

An important finding was that the original 12-item questionnaire required refinement to achieve a more satisfactory measurement model. EDE12 assesses dissatisfaction with body weight or shape; although its removal narrows the direct coverage of body- and weight-related dissatisfaction, related weight- and shape-focused psychopathology remains represented by retained items addressing weight/shape preoccupation, fear of weight gain, desire for weight loss, and the influence of appearance or body weight on self-image. EDE10 assesses the frequency of unusually large eating episodes conditional on loss of control; its removal reduces the specific coverage of the objective quantity aspect of binge eating, while EDE9 retains the core experience of loss of control over eating. Taken together, these findings suggest that the 10-item version retains adequate coverage of the core domains of global eating disorder psychopathology, while modestly narrowing coverage of these two specific manifestations. Importantly, the item-removal decisions were made within the present sample and may therefore reflect sample-specific characteristics. Although the final model demonstrated excellent CFI and TLI and acceptable SRMR, the RMSEA remained slightly above the conventional 0.08 threshold (0.082); therefore, model fit should not be characterized as uniformly excellent. Replication and cross-validation in independent samples are needed before the refined factor structure can be considered established. Previous research has also shown that EDE-Q structures may vary across populations and cultural settings (26, 34), further supporting a cautious interpretation of population-specific psychometric refinements.

Construct validity was supported most clearly by the large positive correlation between the EDE-QS-H and the DESA-6H. Although the questionnaires were designed for different purposes, they assess related aspects of disordered eating: the DESA-6H is an athlete-specific risk screen, whereas the EDE-QS-H measures global eating disorder psychopathology. Kennedy et al. (38) demonstrated the screening performance of the DESA-6, and the Hungarian version has shown satisfactory diagnostic accuracy for identifying athletes at risk (16). The present correlation therefore supports convergence between the measures, but it does not establish diagnostic validity for the EDE-QS-H itself.

Associations between ED psychopathology and multidimensional self-reported interoceptive awareness were heterogeneous and generally small, providing only partial support for the secondary hypothesis. Not-Worrying showed the largest inverse association, while Not-Distracting and Trusting also showed small inverse correlations; Noticing was weakly positively associated with EDE-QS-H scores. In contrast, Attention Regulation, Emotional Awareness, Self-Regulation, and Body Listening were not significantly associated with eating disorder symptoms. This pattern is more consistent with dimension-specific relationships than with a generalized deficit in interoceptive awareness. It is also important to emphasize that the MAIA assesses subjective interoceptive awareness/sensibility rather than objective interoceptive awareness; accordingly, the present findings should not be interpreted as evidence of impaired objective detection of bodily signals. The small effect sizes further indicate that statistically significant associations may have limited practical magnitude and require replication (20, 24, 48, 49).

From a practical perspective, the EDE-QS-H may facilitate preliminary screening and research on eating disorder psychopathology among recreational athletes. Early identification is relevant given the association of disordered eating with adverse health and performance outcomes and with REDs (15). Because the EDE-QS-H is brief and showed promising psychometric performance in this sample, it may be useful to sport psychologists, sports dietitians, clinicians, and researchers as a first-stage measure. However, it should not be used as a stand-alone diagnostic instrument; elevated scores should prompt further assessment using appropriate clinical procedures.

Overall, the present findings provide initial evidence that the refined Hungarian EDE-QS is a reliable brief measure with promising factorial and construct validity in this university-based sample of recreational athletes. The study also suggests that eating disorder symptoms relate selectively, rather than uniformly, to dimensions of self-reported interoceptive awareness. Future studies should cross-validate the 10-item structure in independent samples, evaluate measurement invariance across sex and sport type, establish test–retest reliability, and examine criterion and diagnostic validity against structured clinical assessment.

### 4.1 Strengths

The present study has several methodological strengths that enhance the credibility of the Hungarian adaptation of the EDE-QS. Rather than relying solely on the direct translation of the original questionnaire, the present study applied a psychometrically guided refinement process that resulted in an improved measurement model for the Hungarian version. First, the translation and cultural adaptation followed internationally accepted forward–backward translation procedures and were completed in collaboration with the developer of the original instrument, thereby supporting the linguistic and conceptual equivalence of the Hungarian version. Second, the psychometric evaluation was comprehensive, incorporating confirmatory factor analysis using an estimator appropriate for ordinal data, assessment of internal consistency with both Cronbach’s *α* and McDonald’s *ω*, evaluation of convergent validity through the Average Variance Extracted, and examination of construct validity using theoretically relevant external measures. Finally, by validating the instrument in a sample of physically active athletes, the study provides a practical screening tool for a population in which the early identification of disordered eating is of particular clinical and performance-related importance.

### 4.2 Limitations

Several limitations should be considered when interpreting the findings. First, the study employed convenience sampling from two university settings, which limits representativeness and generalizability to the broader population of Hungarian recreational athletes, as well as to non-athletes, adolescents, older adults, and clinical samples with diagnosed eating disorders. Second, the cross-sectional design precludes evaluation of temporal stability and responsiveness to change; future studies should examine test–retest reliability and longitudinal measurement invariance. Third, although construct validity was supported by associations with conceptually related measures, criterion and diagnostic validity against structured clinical interviews or established diagnostic procedures were not assessed. Fourth, the psychometric refinement from 12 to 10 items was conducted and evaluated within the same sample, creating a potential risk of sample-specific overfitting. The resulting 10-item structure therefore requires replication and cross-validation in independent samples. Furthermore, the removal of EDE10 and EDE12 modestly narrows the coverage of objective binge-eating quantity and explicit body- and weight-related dissatisfaction, respectively, although related aspects remain represented by the retained items. Finally, although a cut-off score has been proposed for the original 12-item EDE-QS, no cut-off value was established or evaluated for the 10-item Hungarian version in the present study. Consequently, the EDE-QS-H should currently be interpreted as a measure of eating disorder symptom severity rather than as a tool for classifying individuals according to a specific screening threshold. Future studies should establish and validate an appropriate cut-off score for the Hungarian version against a suitable diagnostic criterion.

## 5 Conclusion

The present study provides initial evidence that a refined 10-item Hungarian version of the Eating Disorder Examination Questionnaire–Short (EDE-QS-H) has promising psychometric properties in a university-based sample of recreational athletes. The 10-item unidimensional solution demonstrated excellent internal consistency and generally supportive factorial and construct-validity findings, although the borderline RMSEA and sample-specific refinement warrant caution. The EDE-QS-H may be useful as a brief research and preliminary screening measure of eating disorder psychopathology, but the present study does not establish diagnostic validity or clinical utility across broader athlete populations. Independent cross-validation and comparison with structured clinical assessment are needed before broader application is recommended.

## Data Availability

The original contributions presented in the study are included in the article/Supplementary material, further inquiries can be directed to the corresponding author.
