# Supplementary material for: Initial validation of the Hungarian Eating Disorder Examination Questionnaire Short (EDE-QS-H) in a sample of recreational athletes
Source: Front Nutr. 2026 Oct 7;13:1943893. doi: 10.3389/fnut.2026.1943893 (PMC13683691; doi:10.3389/fnut.2026.1943893)
Supplement: Supplementary file 1 [file Supplementary_file_1.docx]

## Kovács et al. 2026

## Supplementary Table S1

### Original English and Hungarian versions of the Eating Disorder Examination Questionnaire–Short (EDE-QS) / EDE-QS-H – Rövid Evészavar Kérdőív

| **Item** | **Original English version (EDE-QS)** | **Hungarian version (EDE-QS-H)** |
| --- | --- | --- |
| 1 | Have you been deliberately trying to limit the amount of food you eat to influence your weight or shape (whether or not you have succeeded)? | Próbálta szándékosan korlátozni az elfogyasztott ételek mennyiségét, hogy befolyásolja testtömegét vagy az alakját (függetlenül attól, hogy sikerült-e vagy sem)? |
| 2 | Have you gone for long periods of time  (e.g., 8 or more waking hours) without eating anything at all in order to influence your weight or shape? | Előfordult, hogy hosszú ideig (pl. 8 vagy több ébren töltött órán át) nem evett semmit a testsúlya vagy alakja megváltoztatása érdekében? |
| 3 | Has thinking about food, eating or calories  made it very difficult to concentrate on things you are interested in (such as working, following a conversation or reading)? | Volt úgy, hogy az ételek, az étkezés vagy a kalóriák körül forgó gondolatai miatt nagyon nehéz volt más dologra koncentrálni (például munka, beszélgetés követése vagy olvasás)? |
| 4 | Has thinking about your weight or shape made it very difficult to concentrate on things you are interested in (such as working, following a conversation or reading)? | Volt úgy, hogy testtömege vagy alakja körül forgó gondolatai miatt nagyon nehéz volt más dologra koncentrálni (például munka, beszélgetés követése vagy olvasás)? |
| 5 | Have you had a definite fear that you might gain weight? | Volt határozott félelme attól, hogy nőni fog testtömege? |
| 6 | Have you had a strong desire to lose weight? | Érzett erős vágyat a fogyásra? |
| 7 | Have you tried to control your weight or shape by making yourself sick (vomit) or taking laxatives? | Próbálta hánytatni magát vagy hashajtót szedni a testtömegének vagy az alakjának szabályozása érdekében? |
| 8 | Have you exercised in a driven or compulsive way as a means of controlling your weight, shape or body fat, or to burn off calories? | Végzett belső késztetésből vagy megszokásból testmozgást a testtömeg, testzsír arány csökkentése, vagy alakjának változtatása, illetve a bevitt kalóriák elégetése érdekében? |
| 9 | Have you had a sense of having lost control over your eating (at the time that you were eating)? | Volt olyan érzése, hogy elvesztette az evés feletti kontrollt, miközben evett? |
| *10* | *On how many of these days (i.e. days on which you had a sense of having lost control over your eating) did you eat what other people would regard as an unusually large amount of food in one go?* | *Az elmúlt 7 napban (azokon a napokon, amikor úgy érezte, hogy elvesztette a kontrollt az evése felett), hányszor evett olyan mennyiségű ételt egy alkalommal, amelyet mások szokatlanul nagy mennyiségűnek tartanának?* |
| 11 | Has your weight or shape influenced how you think about (judge) yourself as a person? | Az önmagáról alkotott képet befolyásolta kinézete vagy testtömege? |
| *12* | *How dissatisfied have you been with your weight or shape?* | *Mennyire volt elégedetlen testtömegével vagy alakjával?* |

**Response options**

**Items 1-10**

| **Score** | **Original English version** | **Hungarian version** |
| --- | --- | --- |
| **0** | 0 days | 0 nap |
| **1** | 1–2 days | 1–2 nap |
| **2** | 3–5 days | 3–5 nap |
| **3** | 6–7 days | 6–7 nap |

**Items 11-12**

| **Score** | **Original English version** | **Hungarian version** |
| --- | --- | --- |
| **0** | Not at all | Egyáltalán |
| **1** | Slightly | Kissé |
| **2** | Moderately | Mérsékelten |
| **3** | Markedly | Határozottan |

Note. This supplementary table presents the complete original English version of the Eating Disorder Examination Questionnaire–Short (EDE-QS) alongside its Hungarian translation (EDE-QS-H) following the forward–backward translation and cross-cultural adaptation process. The original item numbering was retained to facilitate comparison across language versions. As reported in the main manuscript, confirmatory factor analysis and psychometric refinement identified a 10-item Hungarian version (EDE-QS-H), with Items 10 and 12 excluded from the final measurement model because of inadequate psychometric performance. The original English version is reproduced with permission of the original authors.

Each item is scored on a 4-point scale from 0 to 3, with item scores summed to obtain a total score. Higher total scores indicate greater eating disorder psychopathology; no specific clinical cut-off has yet been established for the 10-item Hungarian version (EDE-QS-H).
